# Long-term maintenance of peripheral blood derived human NK cells in a novel human IL-15- transgenic NOG mouse

**DOI:** 10.1038/s41598-017-17442-7

**Published:** 2017-12-08

**Authors:** Ikumi Katano, Chiyoko Nishime, Ryoji Ito, Tsutomu Kamisako, Takuma Mizusawa, Yuyo Ka, Tomoyuki Ogura, Hiroshi Suemizu, Yutaka Kawakami, Mamoru Ito, Takeshi Takahashi

**Affiliations:** 1 Central Institute for Experimental Animals, 3-25-12 Tono-machi, kawasaki-ku, Kawasaki, 210-0821 Japan; 2 Division of Cellular Signaling, Institute for Advanced Medical Research, Keio University School of Medicine, Shinjuku-ku, Tokyo, 160-8582 Japan

## Abstract

We generated a novel mouse strain expressing transgenic human interleukin-15 (IL-15) using the severe immunodeficient NOD/Shi-*scid*-IL-2Rγ^*null*^ (NOG) mouse genetic background (NOG-IL-15 Tg). Human natural killer (NK) cells, purified from the peripheral blood (hu-PB-NK) of normal healthy donors, proliferated when transferred into NOG-IL-15 Tg mice. In addition, the cell number increased, and the hu-PB-NK cells persisted for 3 months without signs of xenogeneic graft versus host diseases (xGVHD). These *in vivo*-expanded hu-PB-NK cells maintained the original expression patterns of various surface antigens, including NK receptors and killer cell immunoglobulin-like receptor (KIR) molecules. They also contained significant amounts of granzyme A and perforin. Inoculation of K562 leukemia cells into hu-PB-NK-transplanted NOG-IL-15 Tg mice resulted in significant suppression of tumor growth compared with non-transplanted mice. Furthermore, NOG-IL-15 Tg mice allowed for engraftment of *in vitro*-expanded NK cells prepared for clinical cell therapy. These cells exerted antibody-dependent cell-mediated cytotoxicity (ADCC) on Her2-positive gastric cancer cells in the presence of therapeutic anti-Her2 antibody, and subsequently suppressed tumor growth. Our results collectively suggest that the NOG-IL-15 Tg mice are a useful model for studying human NK biology and evaluating human NK cell-mediated *in vivo* cytotoxicity.

## Introduction

Mounting evidence has demonstrated that severely immune-deficient mouse strains, including NOD/Shi-*scid*-IL-2Rγ^*null*^ (NOG)^1^ and NOD/LtSz-*scid*-IL-2Rγ^*null*^ (NSG)^2^ mice, allow for long-term engraftment of various xenogeneic tissues. These include human cancer cells^3,4^, multiple human blood cell lineages^5,6^, and some tissues differentiated from human ES/iPS cells^7–9^. Since these engrafted human tissues are maintained autonomously, these “humanized mice” are considered important tools for examining the function and tracing the fates of human tissues *in vivo*.

Natural killer (NK) cells comprise 5–20% of lymphocytes in normal human peripheral blood (PB), and play an important role in eliminating “stressed cells”, such as those infected with viruses and transformed cells^10,11^. The remarkable feature of NK cells is the potent cytotoxic activity they exert on target cells, which has attracted a lot of attention especially in relation to clinical cancer therapies. For example, monoclonal antibodies, which effectively induce NK cell-mediated antibody-dependent cellular cytotoxicity (ADCC), have emerged as one of the most important clinical drugs for treatment of various cancers^12^. Recently, direct infusion of NK cells into leukemia or solid tumor patients has also been attempted^13,14^. To further develop cancer therapies that utilize human NK cells, development of *in vivo* models enabling long-term engraftment of human NK cells is needed.

Since interleukin-15 (IL-15) is indispensable for the differentiation, function, and survival of NK cells^15,16^, NK cells isolated from normal human PB can be stably and functionally maintained in humanized mice if provided with a sufficient amount of human IL-15. When NK cells were induced from umbilical cord blood CD34^+^ HSC in an *in vitro* culture (UCB-NK) and transferred into NSG mice, simultaneous administration of IL-15 induced expansion of NK cells *in vivo* ^17^. Injection of an IL-15 super-agonist, in which IL-15 was bound to soluble IL-15Rα (IL-15/IL-15Rα) into NSG mice engrafted with human-peripheral blood mononuclear cells (PBMCs) activated the human NK population and inhibited acute HIV-1 infection after viral challenge^18^.

In this study, we generated a novel NOG mouse strain that expressed human IL-15 (NOG-IL-15 Tg) and transplanted hu-PB-NK cells from healthy donors into the mice. These hu-PB-NK cells increased in number for the first month and were maintained for the following 2 months in NOG-IL-15 Tg mice, while they disappeared within 2 weeks in conventional NOG mice. The phenotype analysis showed that the expanded human NK cells in NOG-IL-15 Tg mice resembled the original hu-PB-NK cells. We also demonstrated that hu-PB-NK cells in NOG-IL-15 Tg mice could control the growth of K562 tumor cells *in vivo*. Moreover, *in vitro*-expanded highly pure NK cells, which were prepared for clinical cell therapy, were also successfully maintained in NOG-IL-15 Tg mice, but not in NOG-non-Tg mice. Importantly, these cells suppressed tumor growth in the presence of a therapeutic antibody. Our results suggest that NOG-IL-15 Tg mice provide unique opportunities to study and exploit the function of hu-PB-NK cells.

## Results

**Figure 1:**
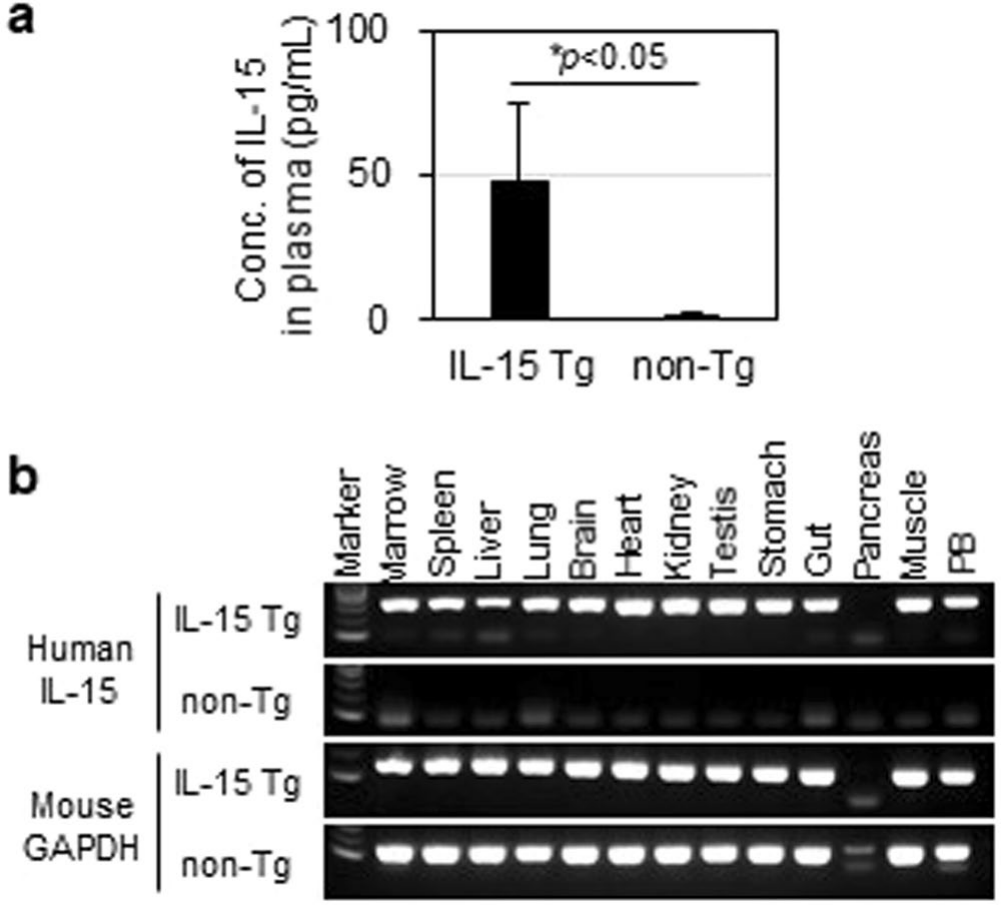
Expression of human interleukin-15 (IL-15) in NOG-IL-15 Tg mice. (**a**) Quantitation of human IL-15. Plasma was prepared from NOG-non-Tg (n = 29) and NOG-IL-15 Tg (n = 126) mice and the level of human IL-15 was determined by enzyme-linked immunosorbent assay (ELISA). Means ± SD are shown. The p-value was obtained using Student’s *t*-test. (**b**) Expression of human IL-15 mRNA in mouse tissues. Total RNA was prepared from various NOG-non-Tg and NOG-IL-15 Tg mouse tissues. After synthesis of cDNA, the presence of human IL-15 and mouse glyceraldehyde-3-phosphate dehydrogenase (GAPDH) was examined by polymerase chain reaction (PCR).

Human IL-15 levels in NOG-IL-15 Tg mouse plasma were about 50 pg/ml (Fig. 1a). Reverse transcription polymerase chain reaction (RT-PCR) analysis also revealed that most mouse tissues contained the transgene-derived transcription (Fig. 1b), suggesting that human IL-15 was produced in a constitutive manner in NOG-IL-15 Tg mice.

**Figure 2:**
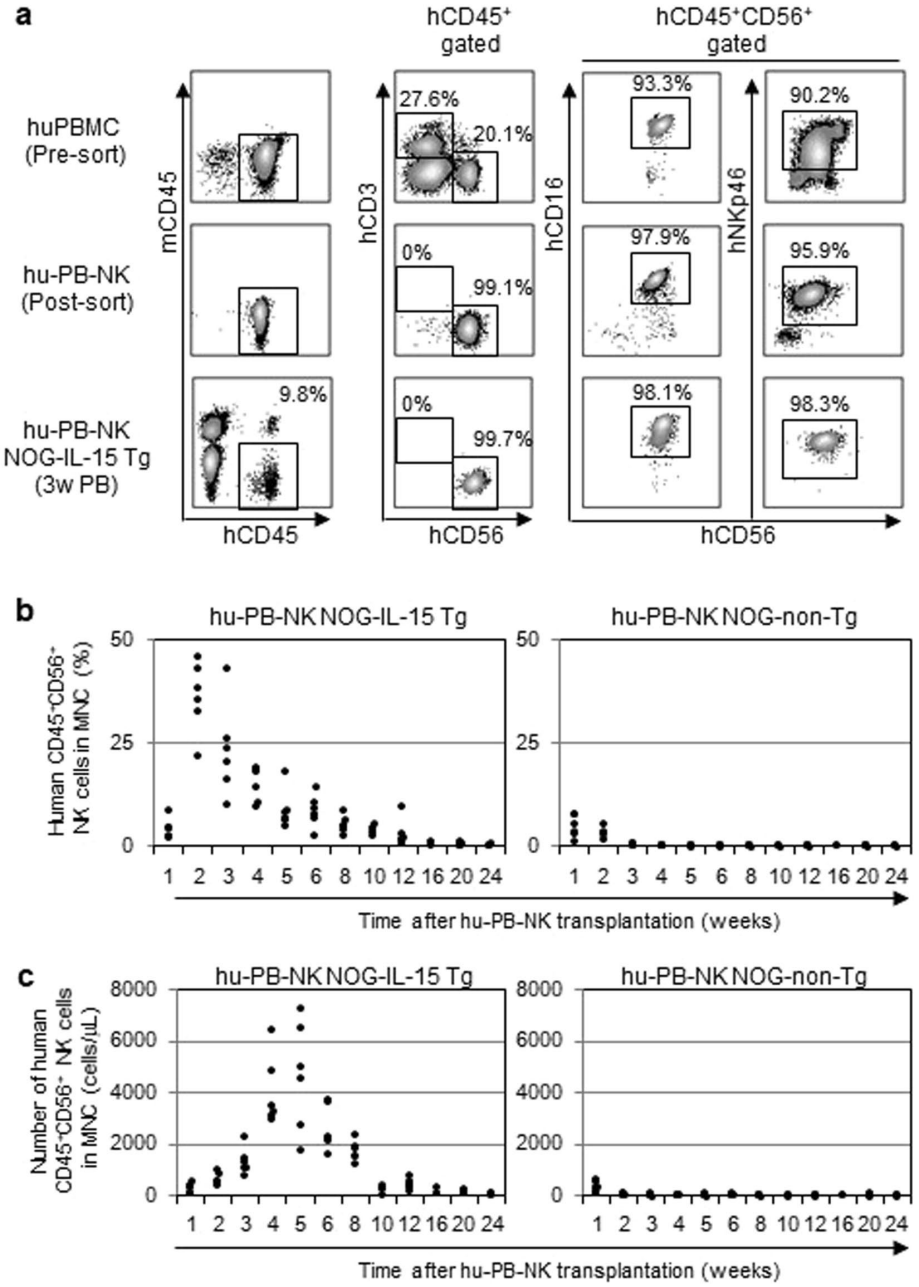
Long-term engraftment of human peripheral blood (PB)-natural killer (NK) cells in NOG-IL-15 Tg mice. (**a**) Fluorescence-activated cell sorting (FACS) plots of human NK cells pre- and post-transfer. Human PB-NK cells from normal donors (top panels) were purified via negative selection by magnetic cell-sorting (MACS). The purified NK cells (1 × 10^6^, intermediate panels) were transferred into NOG-IL-15 Tg mice. PB was collected at 3 weeks after transfer and the presence of hu-PB-NK was analyzed by FACS (bottom panels). A result from four independent experiments is shown. Each experiment used three individual mice and a representative result from one mouse is shown. (**b**,**c**) Expansion of transferred hu-PB-NK cells in NOG-IL-15 Tg mice. Blood was collected and analyzed by FACS every week for 24 weeks after transfer of hu-PB-NK. The frequencies (**b**) and absolute numbers (**c**) of hu-PB-NK cells in mouse PB are shown. Hu-PB-NK cells were freshly isolated from two independent donors. The results from each mouse were plotted (n = 6 for NOG-non-Tg and NOG-IL-15 Tg mice).

The abundant human IL-15 in NOG-IL-15 Tg plasma prompted us to examine whether human NK cells from normal healthy donors (hu-PB-NK) could be maintained upon transplantation. For this experiment, we isolated hu-PB-NK by magnetic cell-sorting (MACS). The majority of the cells were CD56^dull^CD16^+^ NK cells; there were few CD56^hi^CD16^−^ NK cells, reflecting the dominance of the former subpopulation in PB (Fig. 2a). After X-irradiation, we transplanted the purified hu-PB-NK cells into NOG-IL-15 Tg and NOG-non-Tg mice. NK cells could be detected in PB in NOG-IL-15 Tg mice over 12 weeks after transplantation (Fig. 2b,c). In contrast, hu-PB-NK cells disappeared in NOG-non-Tg mice within 10–14 days (Fig. 2b,c), which is consistent with a previous study^19^. The frequency of hu-PB-NK cells rapidly increased for 2 weeks after transplantation and gradually decreased for 6 weeks thereafter (Fig. 2b). The absolute number of hu-PB-NK cells also peaked at around 4–5 weeks after transplantation (Fig. 2c).

**Figure 3:**
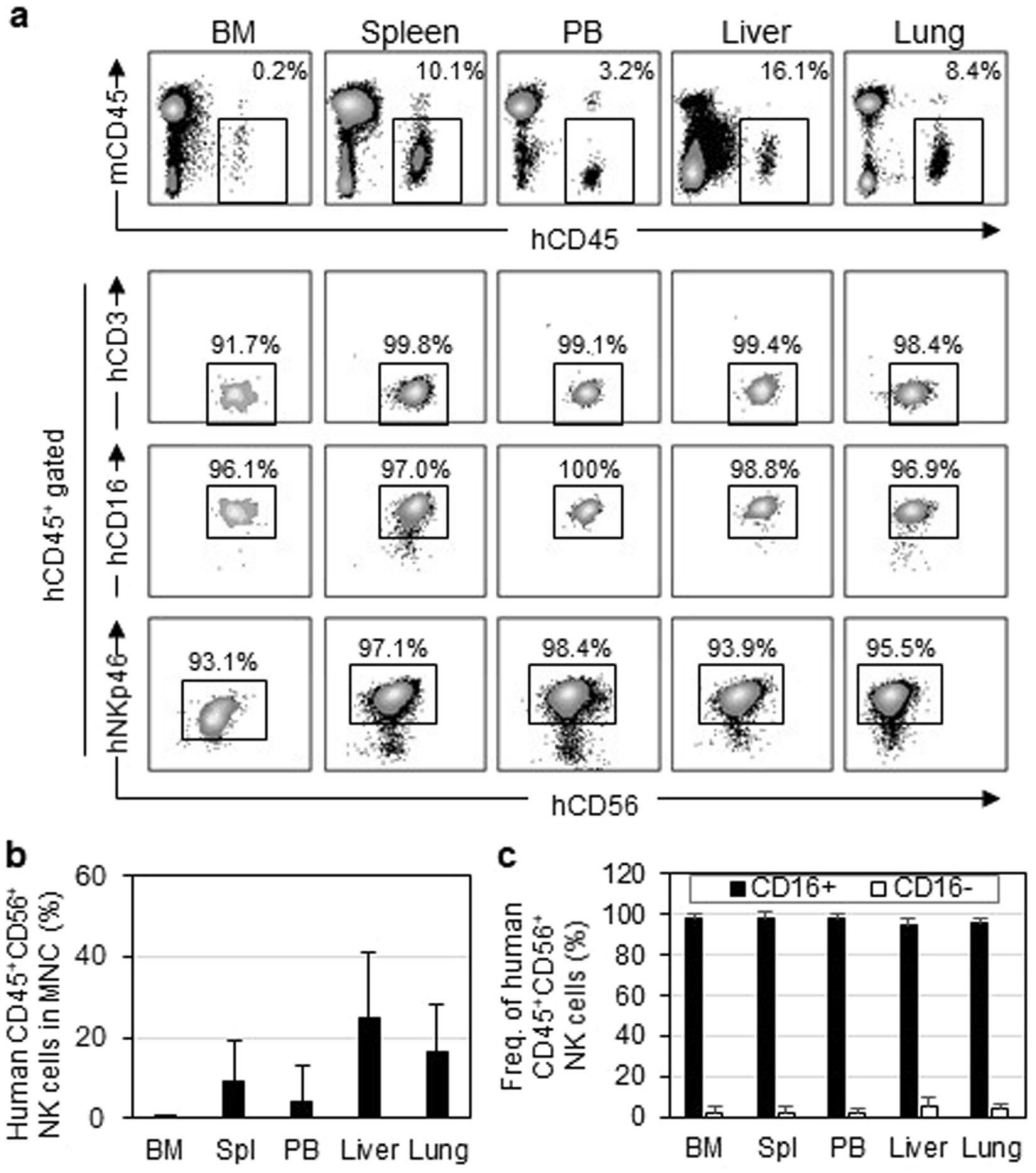
Distribution of human NK cells in NOG-IL-15 Tg mice. (**a**,**b**) Migration of hu-PB-NK cells in various mouse tissues. (**a**) Mononuclear cells from the indicated tissues of hu-PB-NK NOG-IL-15 Tg mice at 8 weeks after NK-transfer were stained with antibodies for indicated antigens and analyzed by FACS. (**b**) Means ± SD of the hu-PB-NK frequencies in each tissue are shown (n = 6). (**c**) Frequencies of CD56^dim^CD16^+^ and CD56^hi^CD16^−^ subpopulations in total CD56^+^ NK cells are shown [n = 6 for bone marrow (BM), spleen, and PB; n = 3 for liver and lung].

Tissue distribution analysis revealed that the expanded human NK cells preferentially migrated into the spleen, liver, and lungs, while some NK cells were found in the bone marrow (BM) (Fig. 3a,b). These cells maintained expression of CD16 and NKp46 and were negative for CD3. The composition of human NK subpopulations was not altered despite the vigorous proliferation. Most cells maintained their original CD56^dull^CD16^+^ phenotype, and few CD56^hi^CD16^−^NK cells appeared (Fig. 3c). Although IL-15 is an indispensable factor for NK cells, it is also important for maintaining T cells, especially memory CD8^+^ T cells^20,21^. However, analyses of mouse PB and spleens demonstrated that only a few human T cells could be detected in all but one mouse, reflecting the stringent purification of human NK cells by multiple-round magnet-sorting (data not shown).

**Figure 4:**
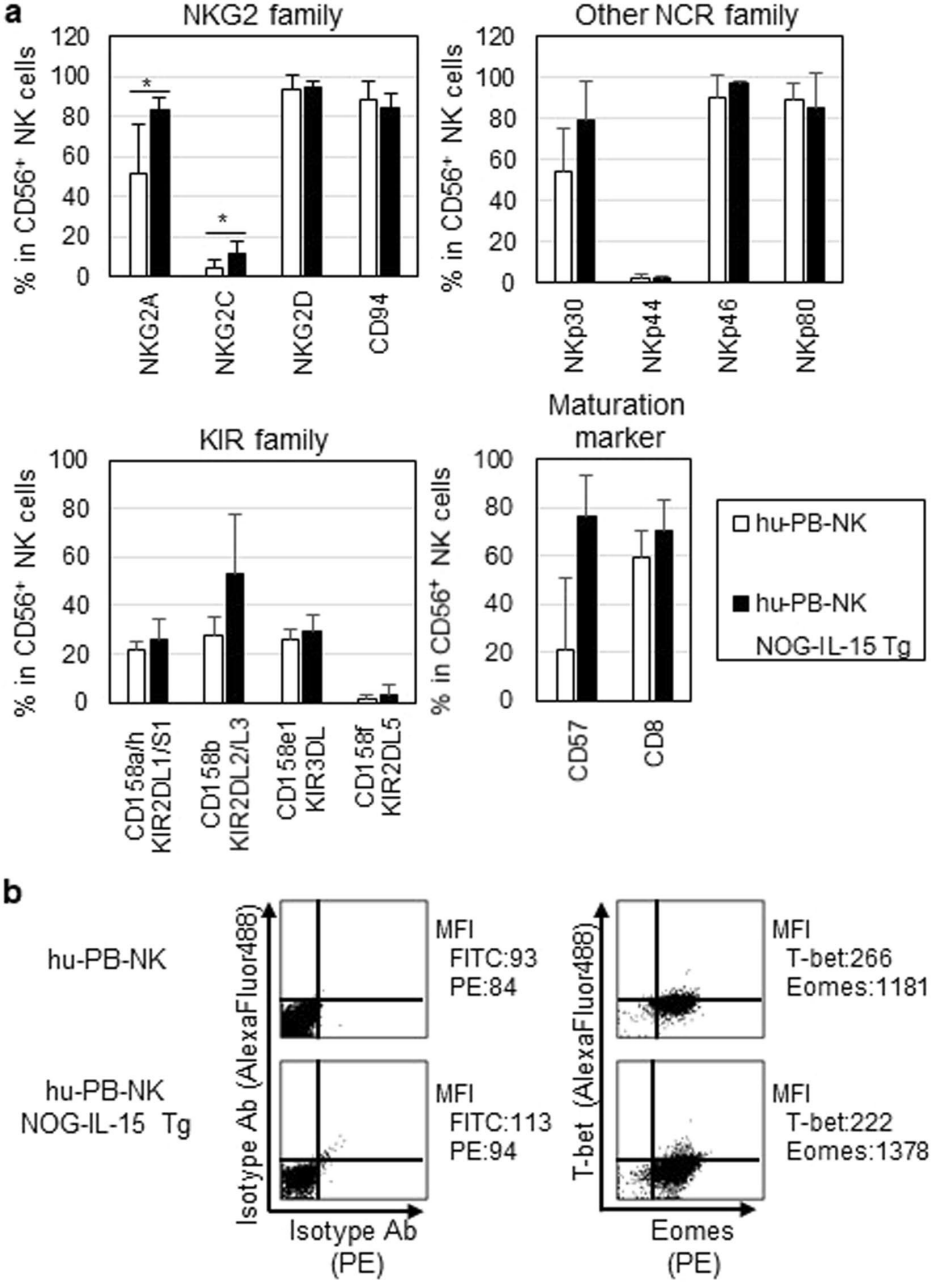
Expression of NK-cell related antigens. (**a**) Expression of NK receptors in hu-PB-NK cells in NOG-IL-15 Tg mice. Freshly isolated hu-PB-NK cells or *in vivo*-expanded NK cells in NOG-IL-15 Tg mice at 6 to 8 weeks after transfer were used for analyses of NK cell receptors, NKG family molecules, natural cytotoxicity receptors (NCRs), and killer cell immunoglobulin-like receptor (KIR) molecules. Expression of CD57 and CD8, maturation markers for NK cells, was also examined. The means ± SD of each fraction are shown (n = 4). Student’s *t*-test was performed to assess statistical significance (*p < 0.05). (**b**) Expression of T-bet and Eomesodermin (Eomes) in human NK cells. The fresh PB-NK cells and *in vivo*-expanded NK cells in NOG-IL-15 Tg mice were derived from different donors. A representative result is shown.

We next examined the phenotypes of hu-PB-NK cells expanded in NOG-IL-15 Tg mice 6–8 weeks after transfer. Natural cytotoxicity receptors (NCRs), including NKp46, the most specific marker for human NK cells, were expressed in expanded hu-PB-NK cells at a level comparable to that in freshly isolated hu-PB-NK cells (Fig. 4a). The expression of various NKG2 family members was also maintained. The frequencies of NK cells expressing NKG2A or NKG2C was significantly higher in expanded hu-PB-NK cells compared with freshly isolated hu-PB-NK (Fig. 4a). However, the expression of killer cell immunoglobulin-like receptors (KIRs) did not differ significantly between expanded hu-PB-NK and freshly isolated hu-PB-NK cells (Fig. 4a). The frequencies of CD57- or CD8-positive fractions, which are markers for the terminal differentiation of NK cells^22^, were not different between these two populations (Fig. 4a). To clarify the stability of cell lineages in the expanded hu-PB-NK cells in NOG-IL-15 Tg mice, we investigated the levels of T-bet and Eomesodermin (Eomes), both of which are indispensable transcription factors for NK cells^23^. Intracellular staining showed that hu-PB-NK cells in NOG-IL-15 Tg mice expressed these transcription factors at comparable levels to fresh hu-PB-NK cells (Fig. 4b). These data collectively suggest that hu-PB-NK cells maintained their immunological features, including the expression of surface antigens and transcription factors, even after massive expansion in NOG-IL-15 Tg mice.

**Figure 5:**
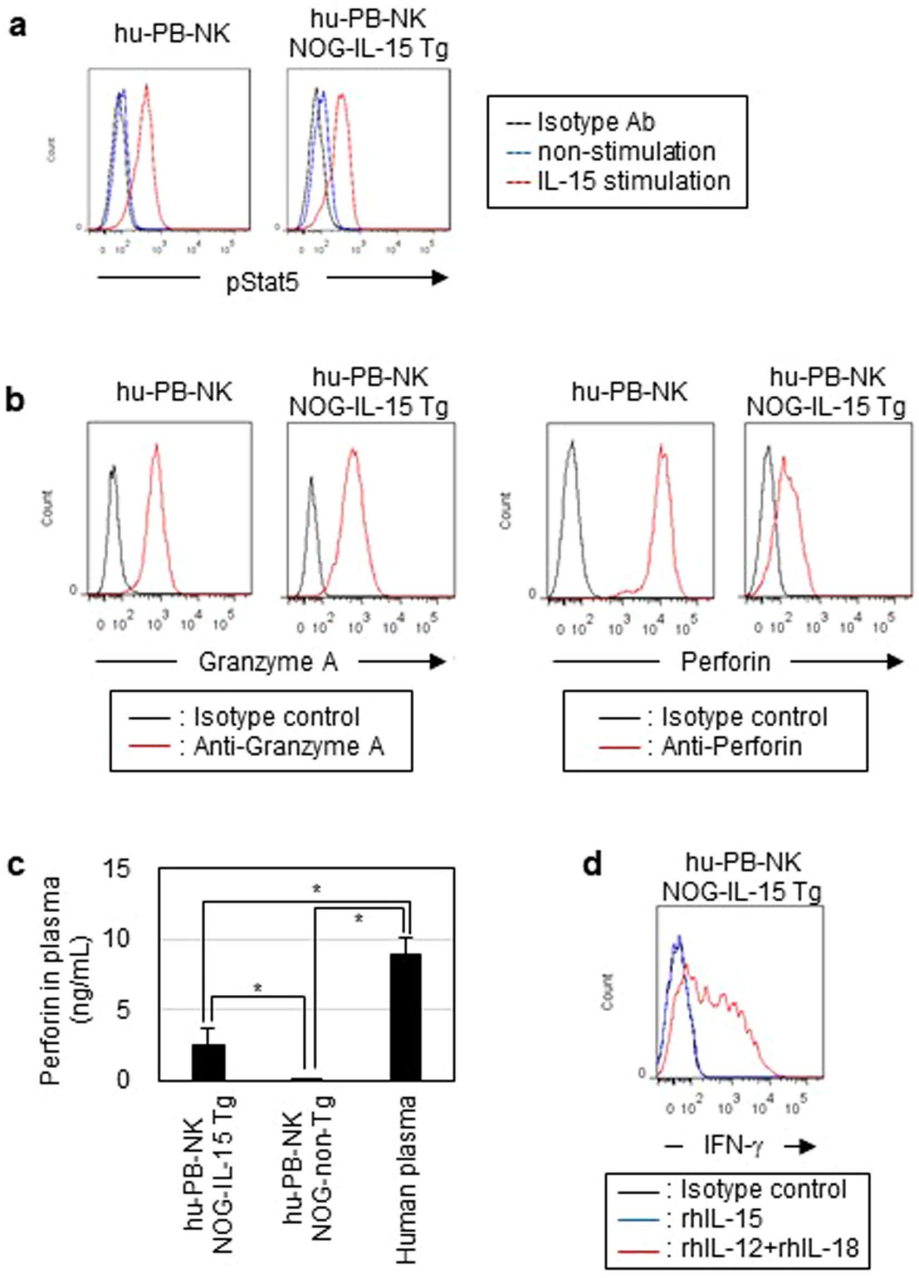
Analysis of functions in *in vivo*-expanded human PB-NK cells. (**a**) Phosphorylation of STAT5. Freshly isolated hu-PB-NK cells and NK cells in NOG-IL-15 Tg mice at 6 weeks after transfer were stimulated with recombinant human IL-15 (2 ng/ml) for 30 min at 37 °C. The cells were analyzed by phosflow. (**b**) Production of granzyme A and perforin molecules. The cells were cultured in the presence of 3 μg/ml breferdin A for 20 h at 37 °C, and subsequently fixed, permeabilized, and stained with indicated antibodies specific for granzyme A or perforin. The fresh PB-NK cells and *in vivo*-expanded NK cells in NOG-IL-15 Tg mice were derived from different donors. (**c**) Quantitation of human perforin in plasma. Plasma was prepared from the PB of NOG-non-Tg (n = 12) or NOG-IL-15 Tg (n = 15) mice at 6 weeks after transfer of hu-PB-NK cells. The plasma from normal human donors (n = 5) was used as a control. Perforin levels were determined by ELISA.The p-value was obtained using Student’s *t*-test (*p < 0.01). (**d**) IFN-γ production of *in vivo*-expanded hu-PB-NK cells. Hu-PB-NK cells prepared from NOG-IL-15 Tg mice at 6 weeks post-transfer were stimulated with recombinant human IL-15 or a combination of human IL-12 and IL-18 in the presence of breferdin A. After culture for 20 h at 37 °C, the cells were intracellularly stained with anti-human IFN-γ. For FACS plots, a representative result from triplicate samples for each staining group is shown. The experiments were repeated twice using different mice and human donors.

We further investigated the function of expanded hu-PB-NK cells. Phosflow experiments showed that stimulation with recombinant IL-15 induced phosphorylation of STAT-5 in hu-PB-NK cells from NOG-IL-15 Tg mice to a similar level as in fresh hu-PB-NK cells (Fig. 5a). We next investigated the cytolytic activity of expanded hu-PB-NK cells in NOG-IL-15 Tg mice. Two major molecules responsible for cytolytic activity in NK cells are granzyme A and perforin. Intracellular staining for these molecules demonstrated that the level of granzyme A in hu-PB-NK cells from NOG-IL-15 Tg mice was comparable to that in fresh hu-PB-NK cells (Fig. 5b). In addition, hu-PB-NK cells from NOG-IL-15 Tg mice expressed high levels of perforin, although levels were lower than in fresh hu-PB-NK cells (Fig. 5c). Analysis of plasma by enzyme-linked immunosorbent assay (ELISA) showed that NOG-IL-15 Tg mice contained a measurable amount of human perforin, while NOG-non-Tg mice did not (Fig. 5c). Stimulation with recombinant IL-12 and IL-18 induced a significant degree of IFN-γ production in hu-PB-NK cells in NOG-IL-15 Tg mice, while almost no production was found in response to stimulation with IL-15 alone. The superior induction of IFN-γ production by IL-12 and IL-18 to IL-15 is consistent with a previous report (Fig. 5d)^24^.

**Figure 6:**
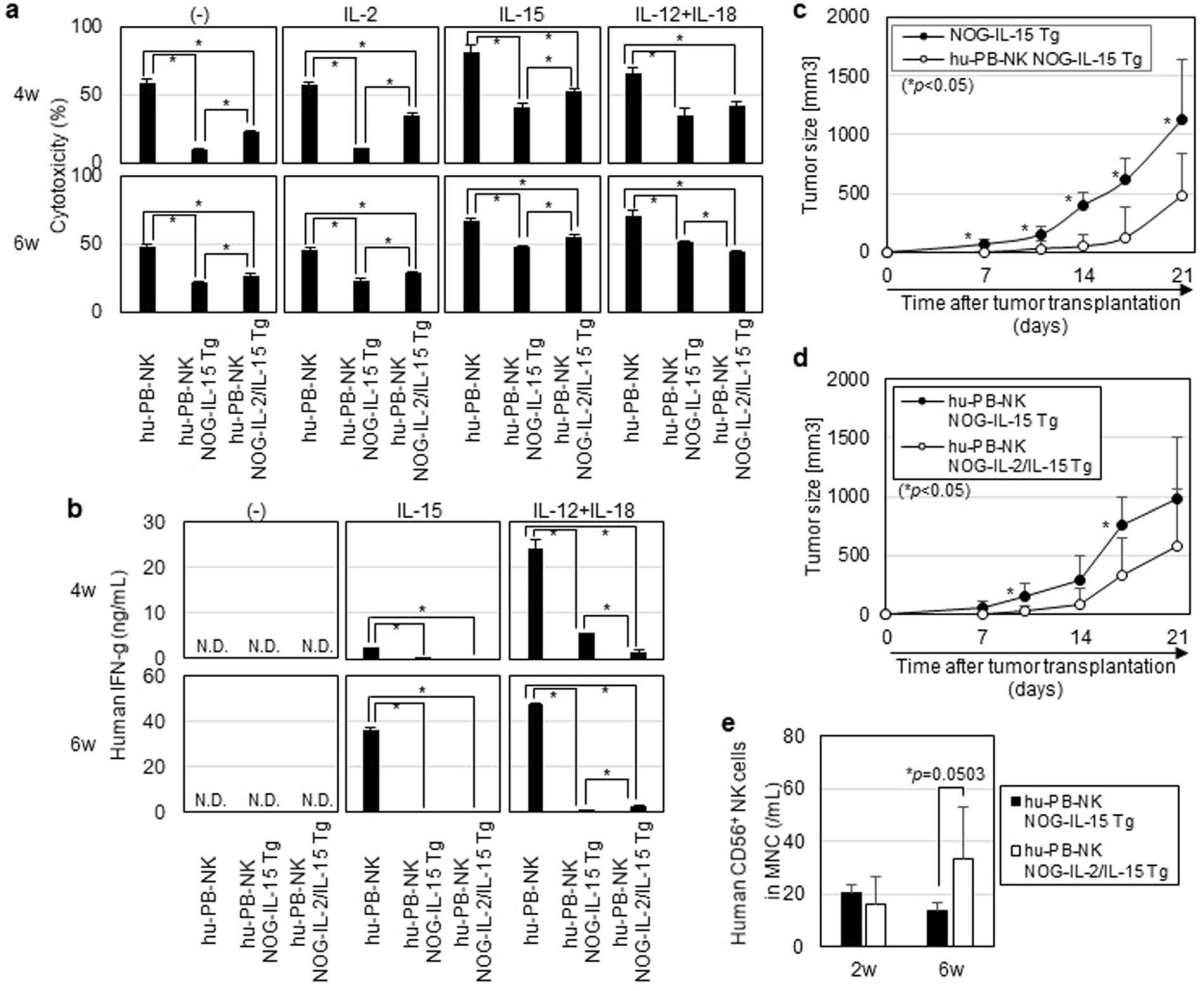
Cytotoxicity of human PB-NK in NOG-IL-15 Tg mice. (**a**) *In vitro* CTL assay. Hu-PB-NK cells from NOG-IL-15 Tg or NOG-IL-2/IL-15 double Tg mice were purified at 4 or 6 weeks post-transfer and cultured for 48 h in the presence of the indicated cytokines. Freshly isolated hu-PB-NK cells were used as a control. The activated NK cells were co-cultured with K562 as a target at a 10:1 ratio for 4 h. The amount of lactate dehydrogenase (LDH) released in the supernatants was measured and CTL activity was calculated using the following formula: Cytotoxicity(%) = [(NK + K562 co-culture)_OD490_ − (K562 culture) _OD490_]/[(K562 Triton-X100-lysed) _OD490_ − (K562 culture) _OD490_]. The fresh PB-NK cells and *in vivo*-expanded NK cells in NOG-IL-15 Tg mice were derived from different donors. Averages from triplicate wells were used for the calculation. A representative result from two independent experiments is shown. The p-value was obtained using Student’s *t*-test (*p < 0.05). (**b**) Production of IFN-γ by NK cells. The supernatants from the 48 h *in vitro* culture after cytokine stimulation, which were described in (**a**), were used for quantitation of IFN-γ by ELISA. Averages from triplicate wells were indicated. A representative result from two independent experiments is shown. The p-value was obtained using Student’s *t*-test (*p < 0.05). N.D. means not detectable. (**c**) Suppression of K562 by hu-PB-NK cells in NOG-IL-15 Tg mice. K562 was subcutaneously inoculated into NOG-IL-15 Tg mice (n = 4) at 3 weeks after hu-PB-NK-cell transfer. Tumor growth was monitored periodically. NOG-IL-15 Tg mice without hu-PB-NK inoculation (n = 4) were used as controls. The results from two independent experiments are shown. *Indicates statisitical significance and the p-value was obtained using Student’s *t*-test. (**d**) Enhanced suppression of tumor growth in NOG-IL-2/IL-15 double Tg mice. Tumor growth was compared between NOG-IL-15 Tg (n = 5) and NOG-IL-2/IL-15 double Tg mice (n = 5). The experiments were conducted as described in (**c**). The results from two independent experiments are shown. The p-value was obtained by Student’s *t*-test. (**e**) Engraftment of hu-PB-NK cells in IL-2/IL-15 double Tg mice. PB was collected from mice used in (**d**) at 2 and 6 weeks post-NK cell transfer and analyzed by FACS. The p-value was obtained using Student’s *t*-test.

I*n vitro* cytolytic (CTL) activity of the expanded hu-PB-NK cells in NOG-IL-15 Tg mice was evaluated using a K562 cell line, which is susceptible to NK cell-mediated cell lysis. While fresh hu-PB-NK cells from normal donors exhibited high CTL activity, hu-PB-NK cells in NOG-IL-15 Tg mice showed modest activity (Fig. 6a). The reduction of CTL activity was already evident at 4 weeks after transplantation. *In vitro* stimulation of hu-PB-NK cells from NOG-IL-15 Tg mice with recombinant IL-2, IL-15, or combination of IL-12 and IL-18 significantly restored the killing activity (Fig. 6a). We measured the level of IFN-γ in the supernatants after the cytokine stimulations (Fig. 6b). IL-15 stimulation induced a significant amount of IFN-γ production in hu-PB-NK cells from normal donors, while a negligible level in hu-PB-NK cells in NOG-IL-15 Tg mice. As shown in a previous report^24^, IL-12 and IL-18 stimulation was more potent than IL-15 in IFN-γ production in normal hu-PB-NK cells (Fig. 6b). Accordingly, *in vitro* culture of hu-PB-NK cells from NOG-IL-15 Tg mice in the presence of IL-12 and IL-18 induced a significant amount of IFN-γ production, but the protein level was much lower than that in hu-PB-NK cells from normal donors (Fig. 6b).

We also investigated whether hu-PB-NK cells in NOG-IL-15 Tg mice could suppress tumor growth *in vivo*. K562 was subcutaneously inoculated into NOG-IL-15 Tg mice at 3 weeks post-hu-PB-NK transplantation. Tumor growth was significantly delayed compared with that in non-transplanted NOG-IL-15 Tg mice (Fig. 6c).

Previous studies have shown that administration of an IL-2 mutant (H9, superkine) could reverse NK cell ‘anergy’ *in vivo* ^25,26^, which is induced in MHC-I-deficient tumor environments. Thus, we suspected that the presence of IL-2 during the expansion of hu-PB-NK cells in NOG-IL-15 Tg mice could maintain NK cell activity. To test this hypothesis, we generated NOG-IL-2/IL-15 double Tg mice and transplanted them with human hu-PB-NK cells. To inhibit the rapid expansion of a few contaminating human T cells, we administered an anti-CD52 monoclonal antibody (mAb). One time administration of anti-CD52 mAb was sufficient to eliminate the human T cells (Supplementary Fig. S2). Hu-PB-NK-engrafted NOG-IL-2/IL-15 double Tg mice were further challenged with K562, and the tumor growth was monitored for 3 weeks. Tumor growth was significantly delayed in NOG-IL-2/IL-15 double Tg mice compared to NOG-IL-15 Tg mice (Fig. 6d). The number of engrafted hu-PB-NK cells was higher in NOG-IL-2/IL-15 double Tg mice compared to NOG-IL-15 Tg mice (Fig. 6e), demonstrating that IL-2 and IL-15 synergistically support the expansion of hu-PB-NK cells.

To determine whether IL-2 improved CTL activity, we measured *in vitro* CTL activity in hu-PB-NK cells in NOG-IL-2/IL-15 double Tg mice (Fig. 6a). Hu-PB-NK cells in NOG-IL-2/IL-15 double Tg mice showed slightly but significantly higher CTL activity than those in NOG-IL-15 Tg mice with or without IL-15 stimulation (Fig. 6a). Interestingly, CTL activity in hu-PB-NK cells in NOG-IL-2/IL-15 double Tg mice after IL-12 and IL-18 stimulation was comparable or even lower than that in NOG-IL-15 Tg mice (Fig. 6a). We also investigated IFN-γ production after cytokine stimulation (Fig. 6b). IL-15 did not induce a detectable amount of IFN-γ in hu-PB-NK cells in NOG-IL-2/IL-15 double Tg mice, while IL-12 and IL-18 stimulation induced a low amount of IFN-γ. We also measured the amount of granzyme A and perforin in the hu-PB-NK cells. Intracellular staining showed no significant differences between the two strains (Supplementary Fig. S3). These results suggest that the anti-tumor activity *in vivo* in NOG-IL-2/IL-15 double Tg mice were most likely due to the increased number of hu-PB-NK cells.

Killing of K562 through direct recognition by hu-PB-NK cells in NOG-IL-15 Tg mice suggested that they could also exert ADCC activity on tumor cells. For this experiment, we used L428, a human CCR4^+^ Hodgkin’s lymphoma cell line, and a therapeutic anti-CCR4 antibody (mogamulizumab; Poteligeo)^27^. After confirming the expansion of hu-PB-NK cells, L428 cells were transplanted and the anti-CCR4 antibody was administered. Unexpectedly, tumor growth was not suppressed in this experimental setting even in the presence of human NK cells (Supplementary Fig. S4).

**Figure 7:**
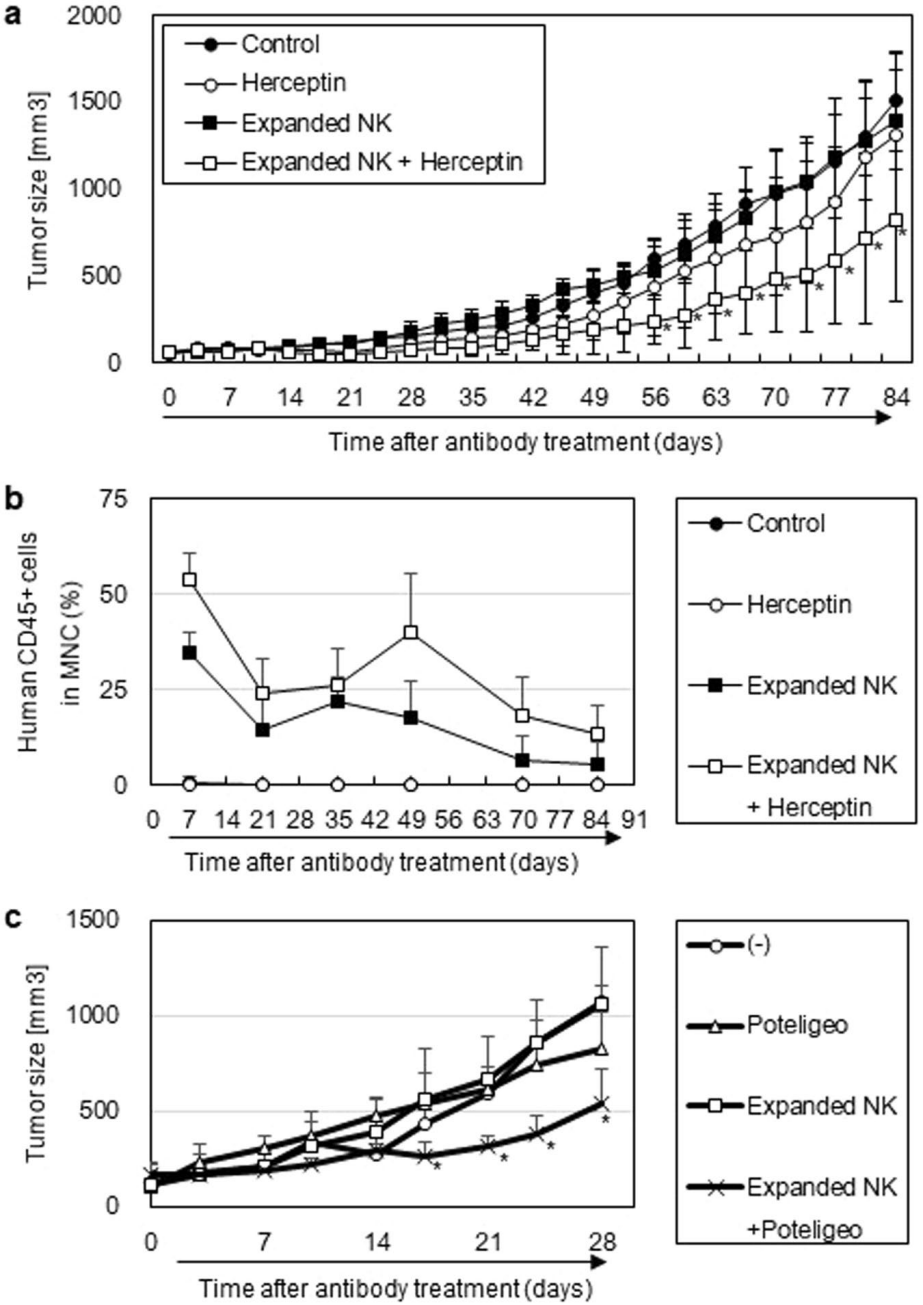
*In vivo* antibody-dependent cell-mediated cytotoxicity (ADCC) activity in hu-PB-NK in NOG-IL-15 Tg mice. (**a**) Tumor suppression by *in vitro*-expanded hu-PB-NK cells and anti-Her2 treatment. Her2-positive NCI-N87 cells, a gastric-cancer cell line, were inoculated into X-irradiated-NOG-IL-15 Tg mice. Hu-PB-NK *in vitro-*cultured cells were transplanted with or without anti-Her2 antibody, as described in the Methods and Supplementary Fig. S5. Tumor growth (mean ± SD) is shown. *Indicates statisitical significance detected by Tukey’s multiple comparisons test with 95% confidence interval. (**b**) Persistence of transfused human NK cells in NOG-IL-15 Tg mice over the experimental period. (**c**) Tumor suppression by *in vitro*-expanded hu-PB-NK cells and anti-CCR4 treatment.NOG-IL-15 Tg mice were X-irradiated and subsequently inoculated with L428 (5 × 10^6^). *In vitro*-expanded hu-PB-NK cells (6 × 10^6^) were transplated 1 week after tumor inoculation and administration of anti-CCR4 antibody (100 μg) or control antibody was started. The antibody treatment were repeated twice a week for up to 4 weeks. Tumor size in NOG-IL-15 Tg (n = 3) or hu-PB-NK NOG-IL-15 Tg mice (n = 3), which were treated with anti-CCR4 antibody or control antibody, was monitored for 4 weeks. *Indicates statisitical significance between the non-treated group and the combined therapy received group. The p-value was obtained using Student’s *t*-test (*p < 0.05).

Thus, we sought an alternative method to monitor *in vivo* ADCC activity using NOG-IL-15 Tg mice, and utilized *in vitro-*expanded human NK cells as effector cells. The protocol for NK cell culture was developed for clinical cell therapy and allows for a roughly 4,000-fold expansion of human NK cells. These cells maintained expression of NKG2D and CD16 and were highly cytotoxic^14^. When these cultured NK cells were transferred into NOG-non-Tg mice, they were poorly maintained. In contrast, these cells were maintained in NOG-IL-15 Tg mice (data not shown). We exploited this system for evaluation of ADCC activity. At first, NOG-IL-15 Tg mice were inoculated with NCI-N87, a human gastric cancer cell line expressing Her2^28^. The mice were subsequently transplanted with the *in vitro-*expanded NK cells, with or without an anti-Her2 antibody (trastuzumab; Herceptin) (Supplementary Fig. S5). Tumors grew in the non-treated mice, the NK cell-transplanted group, and in the antibody-treated mice. In contrast, tumor growth was significantly suppressed in the mouse group that received NK cell and anti-Her2 combination therapy (Fig. 7a). The transferred NK cells were detected in NK cell-transplanted groups irrespective of the anti-Her2 treatment (Fig. 7b). We also tested this *in vivo* ADCC system using the combination of L428 and the anti-CCR4 antibody. L428 was transplanted in NOG-IL-15 Tg mice and subsequently *in vitro-*expanded NK cells were transplanted. The anti-CCR4 antibody was given twice a week for 4 weeks. Only the mouse group which received both NK cells and anti-CCR4 antibody therapy showed significant suppression in tumor growth (Fig. 7c).

## Discussion

In this study, we established a novel NOG-IL-15 Tg strain, which provides unique experimental systems in which hu-PB-NK cells can be maintained for up to 3 months. This is the most distinguishing characteristic of NOG-IL-15 Tg mice compared to other models, including conventional NOG and NOG-IL-2 Tg mice, in which human NK cells are predominantly differentiated after HSC transplantation^27^. Hu-PB-NK cells disappeared within 2 weeks of transplantation in NOG mice, as described previously^19^. In NOG-IL-2 Tg mice, engraftment of PB-NK cells was not supported in spite of the capacity to induce human NK cells from HSC (Supplementary Fig. S6). These results are in line with the specific requirements of IL-15 for NK cell survival.

The level of IL-15 in NOG-IL-15 Tg mice was 20~100 pg/ml, which was significantly higher than normal human serum (~1 pg/ml) and may be a cause for concern regarding the strong influence of NK cells beyond the physiological level. However, considering that similarly high levels of IL-15 were detected in lympho-depleted patients^29^, the levels in NOG-IL-15 Tg mice may be not unusually high. In addition, it is possible that the amount of IL-15 does not predict the biological influence of human NK cells, since it is a heterodimer consisting of IL-15 and IL-15Rα^30,31^ on myeloid cells that stimulate IL-15Rβ and γc complexes in NK cells. Thus, in NOG-IL-15 Tg mice, a human IL-15/mouse IL-15Rαchimeric complex would stimulate human NK cells. In this situation, the amounts of human IL-15 and mouse IL-15Rα may restrict the signal intensity of human NK cells.

The composition and phenotypes of hu-PB-NK cells were maintained in NOG-IL-15 Tg mice before transplantation and after expansion. Human NK cells consist of CD56^dull^CD16^+^ and CD56^hi^CD16^−^ populations. CD56^dull^CD16^+^ cells constitute the largest proportion of hu-PB-NK cells, while CD56^hi^CD16^−^ cells are usually found only in secondary lymphoid^32^. The functions of the two populations are also different^33,34^. CD56^dull^CD16^+^ cells are cytotoxic, while CD56^hi^CD16^−^ cells are involved in immune regulation through cytokine production. The relationship between these two populations is still a matter of debate, and it is not known whether the two populations are independent or if they represent different stages in a common differentiation pathway series. We demonstrated that hu-PB-NK cells, which contained more than 98% CD56^dull^CD16^+^ cells, did not produce CD56^hi^CD16^−^ cells in NOG-IL-15 Tg mice, even after proliferation and migration into different tissues. These results suggest that CD56^dull^CD16^+^ cells are independent from CD56^hi^CD16^−^ cells. On the contrary, CD56^hi^CD16^−^ populations may be able to differentiate into CD56^dull^CD16^+^ cells^35^. The isolation of a sufficient number of CD56^hi^CD16^−^ NK cells and transplantation into NOG-IL-15 Tg mice would answer this question.

Reduction of *in vitro* CTL activity and IFN-γ production in hu-PB-NK cells in NOG-IL-15 Tg mice was not predicted. However, this is reminiscent of several mouse studies showing that NK cells are desensitized in various experimental settings. The first example was reported by Elpelk *et al*., who demonstrated that chronic treatment of mice with IL-15/IL-15Rα complexes resulted in the accumulation of mature NK cells with impaired function^36^. Most NK cells were CD11b^+^CD27^-^KLRG1^+^ senescent cells with altered expression of NK cell receptors, specifically downregulation of activating receptors and upregulation of inhibitory receptors. They suggested that chronic stimulation by IL-15/IL-15Rα initially induced NK cell activation and proliferation, but in turn prevented induction of apoptosis in expanded NK cells, resulting in the accumulation of senescent NK cells. It is possible that hu-PB-NK cells in NOG-IL-15 Tg mice represent a similar senescent population. Indeed, alteration in the expression of some NKG2 receptors would agree with this mechanism. In addition, NK cells are rendered “anergic” under MHC-deficient conditions, as reported in MHC-I deficient mice^37^. This type of function loss was also demonstrated in NK cells in tumor environments where the expression of MHC-I is often lost^26^. Since there are no mouse homologues for HLA-C that interact with KIRs on human NK cells, “anergy” might be induced in NOG-IL-15 Tg mice. Exhaustion of NK cells may also occur. Adoptive transfer experiments showed that NK cells lost their killing activity upon homeostatic proliferation and tumor exposure, and that reduction of T-bet and Eomes expression was associated with loss of function^38,39^. Our intracellular staining of T-bet and Eomes in hu-PB-NK cells in NOG-IL-15 Tg mice demonstrated that the T-bet levels became slightly, but not statistically significantly, lower, while the Eomes level was maintained. Thus, it is not likely that desensitization of NK cells in NOG-IL-15 Tg mice is directly related to this type of NK cell-exhaustion. However, these three mechanisms are not always mutually exclusive. IL-15 rich, HLA-C absent, and immunodeficient environments in NOG-IL-15 Tg mice can activate molecular programs in human NK cells. Therefore, a comparison between senescent, anergic, or exhausted mouse NK cells and human NK cells in NOG-IL-15 Tg mice through gene expression profiling and epigenetic studies will be necessary in future works.

The *in vivo* experiments examining the cytotoxic activity of hu-PB-NK cells in NOG-IL-15 Tg mice seemed to be contradictory. When K562 cells were used, hu-PB-NK cells could control tumor growth, although they could not totally eradicate the tumor cells. In contrast, when L428 was used in conjunction with an anti-CCR4 antibody, no tumor suppression was seen. Given the high sensitivity of K562 cells, NK-cell mediated cell lysis, and partial suppression, but not rejection, of tumor growth may suggest that *in vivo*-expanded NK cells had only weak cytotoxic activity, which was consistent with *in vitro* results. Although the mechanisms responsible for the compromised NK function in NOG-IL-15 Tg mice remain to be elucidated, an important question is whether hu-PB-NK cells can be rejuvenated or activated in NOG-IL-15 Tg mice *in vivo*. Our experiments using NOG-IL-2/IL-15 double Tg mice demonstrated synergistic effects on hu-PB-NK cells. Although the CTL activity and IFN-γ production in hu-PB-NK cells was not remarkably restored in NOG-IL-2/IL-15 double Tg mice, the superior increase in the number of hu-PB-NK cells in NOG-IL-2/IL-15 double Tg mice compared with NOG-IL-15 Tg mice resulted in better tumor control *in vivo*, indicating that using double transgenic mice could be an interesting approach.

We found that NOG-IL-15 Tg mice could maintain not only freshly isolated human hu-PB-NK cells, but also *in vitro*-expanded NK cells. Although multiple administrations were necessary to maintain cellularity, this may be due to the terminal differentiation of cells by vigorous proliferation *in vitro*. Nevertheless, the suppression of gastric cancer tumors or leukemia in NOG-IL-15 Tg mice by *in vitro*-expanded NK cells in combination with anti-Her2 or anti-CCR4 antibody treatment, respectively suggests that these cells had potent cytotoxicity. An important question would be the differences between human NK cells in NOG-IL-15 Tg mice and *in vitro*-expanded human NK cells. Both of them experienced vigrous proliferation by cytokine stimulation. Nevertheless, they have different CTL activity, *i*.*e*. Human NK cells expanded in NOG-IL-15 Tg mice had reduced CTL activity, while *in vitro*-expanded human NK cells maintained it. Thus, these NK cells might represent different two distinct NK-derived populations. This should be clarified in the future experiments by using gene expression profiling. In contrast, there is another possibility that these two NK cells are not very different. Considering the induction of tolerance in human PB-NK cells in NOG-IL-15 Tg mice, it is possible that *in vitro*-expanded NK cells are also compromised by the same mechanisms. This possibility cannot be tested, however, due to the short term maintenance of *in vitro*-expanded NK cells in NOG-IL-15 Tg mice. Practically, NOG-IL-15 Tg mice and *in vitro*-expanded human NK cells have a good affinity. Although the utility of those cultured NK cells needs to be tested *in vivo* before clinical applications, there have been no good animal models where the performance of human NK cells could be examined *in vivo*. Our NOG-IL-15 Tg or NOG-IL-2/IL-15 double Tg mice will be useful for the verification of those cultured human NK cells.

Collectively, we established that NOG-IL-15 Tg mice enable long-term engraftment of human hu-PB-NK cells *in vivo*. This model could be useful for studying various immunological features of human NK cells. Graft-versus-leukemia (GVL) effects of human NK cells on tumor cells, and the ADCC-inducing activity of drug candidates, could be evaluated *in vivo*, which would help us develop novel therapeutic protocols for malignant diseases.

## Methods

### Mice

NOG, NOD/ShiJic-IL-2Rγ^*null*^ (NOD-IL-2Rγ^*null*^), and NOG-IL-2 Tg [formally NOD.Cg-*Prkdc* ^*scid*^ *il2rg* ^*tmlSug*^ *Tg (CMV-IL2)4-2Jic*/Jic] mice^27^ were used in this study. These strains were maintained in the Central Institute for Experimental Animals (CIEA) under specific pathogen-free conditions.

To generate human IL-15-expressing transgenic NOG mouse strains, we obtained the human IL-15 cDNA clone (#1866)^40^ from RIKEN BRC (Tsukuba, Japan). We then constructed the transgene DNA containing the human IL-15 coding region, whose signal peptide sequence was replaced with those of the human IL-2 gene for improving secretion (Supplementary Fig. S1) under the control of the CMV promoter. The fragment was microinjected into fertilized eggs of NOD-IL-2Rγ^*null*^ mice. Founder mice were backcrossed with NOG mice to obtain NOG-IL-15 Tg [formally NOD.Cg-*Prkdc* ^*scid*^ *il2rg* ^*tm1Sug*^ *Tg (CMV-IL15)1 Jic*/Jic] mice. NOG-IL-2/IL-15 double transgenic mice were produced by mating NOG-IL-15 Tg with NOG-IL-2 Tg mice in our facility.

All experiments were performed in accordance with institutional guidelines (11004, 14038 R), which were approved by the Animal Experimentation Committee of CIEA.

### Measurement of interleukin-15

To quantify the amount of human IL-15 protein in the NOG-IL-15 Tg plasma, PB of 5-week-old mice was collected using heparin (Novo-heparin; Mochida Pharmaceutical Co., Tokyo, Japan) and the IL-15 levels in the plasma were determined by ELISA (BioLegend, San Diego, CA, USA).

### Reverse transcription polymerase chain reaction

Mouse tissues were homogenized in ISOGEN (Nippon Gene, Toyama, Japan). Total RNA was prepared according to the manufacturer’s instructions. The first strand of cDNA was synthesized by SuperScript III (Invitrogen, Carlsbad, CA, USA) after DNase treatment. The human IL-15 or mouse glyceraldehyde-3-phosphate dehydrogenase (GAPDH) genes were amplified by PCR using the following specific primers:

Human IL-15 forward primer: 5′-CTGGGTGAATGTAATAAGTGATTTG-3′

Human IL-15 revers primer: 5′-TTTTTCCTCCAGTTCCTCACA-3′

Mouse GAPDH forward primer: 5′-TGTGTCCGTCGTGGATCTGA-3′

Mouse GAPDH revers primer: 5′-TTGCTGTTGAAGTCGCAGGAG-3′

The PCR products were detected by electrophoresis in 2% agarose gels.

### Purification and transplantation of human NK cells

Human PB was obtained from multiple healthy volunteers after acquiring their informed consent. Human PBMCs (hPBMCs) were prepared by density centrifugation on Ficoll (Lymphoprep; Axis-Shield, Oslo, Norway). For separation of human CD56^+^ NK cells from hPBMCs, we used a human NK isolation kit (Miltenyi Biotec, Bergisch Gladbach, Germany) in combination with biotin-conjugated anti-mouse CD45 mAb (BD Biosciences, San Jose, CA, USA). All cells except human CD56^+^ NK cells were magnetically labeled and eliminated twice using a MACS LD column (Miltenyi Biotec). The purity of the isolated human NK cells was usually greater than 95%. We also used human Caucasian PB-derived CD56^+^ NK cells, purchased from AllCells (Berkeley, CA, USA), in some experiments. For transplantation, 8–12-week old mice were X-irradiated (2.5 Gy) (MBR-1505R; Hitachi Medical Corp., Tokyo, Japan) and 1–2 × 10^6^ hu-PB-NK cells suspended in phosphate-buffered saline (PBS) were transplanted intravenously within 24 h. To monitor the engraftment, the mice were bled every week, and the mononuclear cells were analyzed by flow cytometry.

### Preparation of immune cells in tissues of NK cell-transferred mice

PB was collected from an abdominal vein under anesthesia using isoflurane. Blood plasma was separated by centrifugation. BM was flushed from femurs with 1 ml PBS containing 2% fetal bovine serum (FBS). Spleen cells and lymphocytes in the liver were prepared by smashing the tissues between two frosted glass slides, and the tissue debris was removed by nylon mesh. To release cells in the lungs, tissues were incubated with 1 mg/ml collagenase and 50 ng/ml DNase in RPMI 1640 (Gibco, Grand Island, NY, USA) for 30 min at 37 °C, followed by filtration through mesh. Mouse red blood cells (RBCs) were eliminated with RBC lysing solution (Pharm Lyse; BD Biosciences). After washing, the cell pellet was resuspended with PBS + 2% FBS.

### Flow cytometry

Single mononuclear cell suspensions were stained with antibodies for 30 min at 4 °C in the dark. Cells were then washed with fluorescence-activated cell sorting (FACS) buffer (PBS, 0.5% FBS, 0.05% NaN_3_) and resuspended in FACS buffer containing propidium iodide (PI) before being subjected to multicolor flow cytometric analysis by a FACS Canto machine (BD Biosciences). The data were analyzed using FACS Diva software (ver. 6.1.3; BD Biosciences).

The antibodies used were as follows: Alexa Fluor® 488-conjugated anti-human natural killer group 2 membrane C (NKG2C) from R&D Systems (Minneapolis, MN, USA) and anti-human CD159a (NKG2A)-PE and anti-human NKp80-PE (Beckman Coulter, Miami, FL, USA). The following antibodies against human antigens were from BioLegend: fluorescein isothiocyanate (FITC)-conjugated anti-CD8a, anti-CD16, anti-CD158a/h, and anti-CD158e1; phycoerythrin (PE)-conjugated anti-CD56, anti-NKp30, anti-NKp44, anti-NKp46, anti-CD57, anti-NKG2D, anti-CD158b, and anti-CD158f; PE-Cy7-conjugated anti-CD3 and anti-CD56; APC-conjugated anti-CD16 and anti-CD94 monoclonal antibodies; and APC-Cy7-conjugated anti-CD45. The APC conjugated anti-mouse CD45 antibody was from BioLegend.

The absolute number of cells was calculated using fluorescent beads (Flow-Count; Beckman Coulter, Brea, CA, USA) according to the manufacturer’s instructions.

### Cell culture

K562 (human erythromyeloblastoid leukemia cell line) cells were maintained in complete RPMI 1640 medium supplemented with 10% FBS, MEM non-essential amino acids (NEAAs), 50 μM 2-mercaptoethanol (2-ME), 100 U/ml penicillin, and 100 μg/ml streptomycin. NCI-N87 cells, which were established from a liver-metastasized gastric cancer^28^, were cultured in complete RPMI medium with 10% FBS.

### *In vitro* cytotoxicity assay

The cytotoxic activity of human NK cells was measured using K562 as target cells *in vitro*. In some experiments, human NK cells were stimulated with 2 ng/ml IL-2, 2 ng/ml IL-12, 2 ng/ml IL-15 (Miltenyi Biotec), or 2 ng/mL IL-18 (Medical & Biology Laboratories, Nagoya, Japan), either alone or in combination for 48 h before use. These cells were subsequently incubated with K562 for 4 h. The effector/target ratio was 10: 1. The release of lactate dehydrogenase (LDH) into the culture supernatant was measured using a Cytotox96 non-radioactive cytotoxicity assay kit (Promega, Madison, WI, USA). We used OptEIA Human IFN-γ ELISA Kit II (BD Biosciences) for measuring the amount of human IFN-γ in the culture supernatants.

### Intracellular staining

The expression of perforin, granzyme A, and IFN-γ in human NK cells was examined by intracellular staining. Purified human NK cells were stimulated with either PMA/Ionomycin or IL-12/IL-18 for 20 h at 37 °C in the presence of 3 μg/ml brefeldin A. After the cells were stained with antibodies for surface antigens, they were fixed in IC fixation buffer (eBioscience, San Diego, CA, USA) and subsequently permeabilized using a permeabilization wash buffer (BioLegend). These cells were the stained with anti-human granzyme A-FITC, anti-human perforin-PE, or anti-human IFN-γ-PE (BioLegend) in Perm/Fix solution for 30 min at room temperature.

To detect transcription factors in human NK cells, we utilized a fixation buffer in the FOXP3 Fix/Perm Buffer Set (BioLegend). After fixation, cells were permeabilized and stained with anti-human T-bet-Alexa Fluor® 488 (BD Biosciences) and anti-human Eomes-PE (eBioscience), or mouse IgG1-Alexa Fluor® 488 and mouse IgG1-PE, as an isotype control (BD Biosciences), in FOXP3 Perm buffer for 30 min at room temperature.

To detect phosphorylation of STAT5, hu-PB-NK cells from normal donors or splenic NK cells from hu-PB-NK transferred NOG-IL-15 Tg mice were cultured at 37 °C in the presence or absence of recombinant IL-15 for the periods indicated and subsequently fixed with fixation buffer (BD Biosciences). After washing with FACS buffer three times, the cells were resuspended with Perm Buffer III (BD Biosciences) and incubated for 30 min on ice. After washing four times, the cells were stained with Alexa Fluor® 488-labeled anti-STAT5 (pY694) or Alexa Fluor® 488-labeled mouse IgG1 as an isotype control (BD Biosciences), for 60 min at 37 °C. After washing, these cells were resuspended with FACS buffer and analyzed using a FACS Canto machine.

### Quantification of perforin by enzyme-linked immunosorbent assay

Perforin levels in plasma from hu-PB-NK transferred NOG-IL-15 Tg mice were measured using a human perforin-specific ELISA kit (Abcam, Cambridge, UK) at 6 weeks after transplantation.

### Large scale preparation of human natural killer cells *in vitro*

Human NK cells were expanded from PBMCs by Takara Bio (Shiga, Japan). The protocol was described in detail elsewhere^14^. Starting with 50 ml PB, ~1 × 10^9^ pure human NK cells were yielded.

### *In vivo* tumor transplantation model

The *in vivo* killing activity of human NK cells in hu-PB-NK-transferred NOG-IL-15 Tg and NOG-IL-2/IL-15 double Tg mice was investigated by transplantation of K562 tumor cells at 3 weeks post-NK cell transfer. K562 (with 2.5 × 10^5^ in 0.1 ml of PBS) cells were inoculated subcutaneously into the midline of the dorsal region of the mice. Solid tumor size was measured weekly using micrometer calipers and calculated by the following formula: tumor volume [mm^3^] = 1/2 × length (mm) × [width (mm)]^2^.

### *In vivo* antibody-dependent cellular cytotoxicity

The experiment using *in vitro* expanded NK cells was conducted as shown in Fig. S4. NCI-N87 (3 × 10^6^) was subcutaneously inoculated into 2.5Gy-X-irradiated NOG-IL-15 Tg mice 1 week prior to the beginning of the test (day 0). *In vitro* expanded NK cells (99% pure and 1 × 10^7^) and anti-Her2 antibody (10 mg/kg Herceptin; Chugai, Tokyo, Japan) were administered by intravenous and intraperitoneal injection, respectively. Transfer of NK cells was done twice (days 3 and 5). Anti-Her2 antibody was also given (days 5 and 7). The same regimen was repeated once more starting on day 28. Tumor size was measured twice a week over a period of 12 weeks.

L428 (with 5 × 10^6^ in 0.1 ml PBS) cells were subcutaneously inoculated into X-irradiated-NOG-IL-15 Tg mice. After the tumor became visible at 1 weeks post-inoculation, *in vitro*-expanded human NK cells (with 6 × 10^6^ in 0.2 mL PBS) were intravenously transferred and therapeutic anti-human CCR4 antibody (POTELIGEO®; Kyowa-Kirin, Tokyo, Japan) was intraperitoneally injected twice a week for 4 weeks.

## Electronic supplementary material

Dataset 1
