# Supplementary material for: Long-term maintenance of peripheral blood derived human NK cells in a novel human IL-15- transgenic NOG mouse
Source: Sci Rep. 2017 Dec 8;7:17230. doi: 10.1038/s41598-017-17442-7 (PMC5722902; doi:10.1038/s41598-017-17442-7)
Supplement: Supplementary file 1 — Dataset 1 [file 41598_2017_17442_MOESM1_ESM.doc]

# Title**: Long-term maintenance of peripheral blood derived human NK cells in a novel human IL-15- transgenic NOG mouse**

Ikumi Katano^*†^, Chiyoko Nishime^*^, Ryoji Ito^*^, Tsutomu Kamisako^*^, Takuma Mizusawa^*^, Yuyo Ka^*^, Tomoyuki Ogura^*^, Hiroshi Suemizu^*^, Yutaka Kawakami^†^, Mamoru Ito^*^, Takeshi Takahashi^*‡^

^*^  Central Institute for Experimental Animals, 3-25-12 Tono-machi, kawasaki-ku, Kawasaki, 210-0821, Japan

^†^  Division of Cellular Signaling, Institute for Advanced Medical Research, Keio University School of Medicine, Shinjuku-ku, Tokyo, 160-8582, Japan

^‡^ Address corresponding: Takeshi Takahashi, Mamoru Ito, Central Institute for Experimental Animals, 3-25-12 Tono-machi, kawasaki-ku, Kawasaki, 210-0821, Japan. Phone: +81-44-201-8537; Fax: +81-44-201-8541; E-mail: takeshi-takahashi@ciea.or.jp

**Supplementary Figure legends**

**Figure S1. Human IL-15 transgene construct.**

The signal peptide of the human IL-2 gene was fused to the human IL-15 cDNA. The fragment consisted of the CMV promoter, the IL-15 cDNA and polyA was purified after PvuII digestion and used for microinjection.


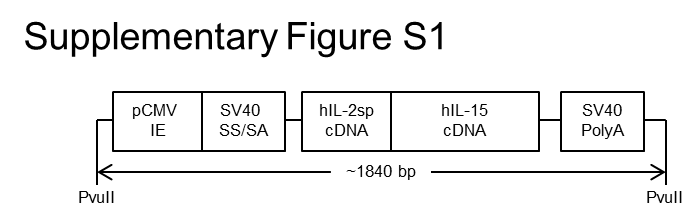


**Figure S2. *in vivo* T cell depletion by anti-CD52 monoclonal antibody treatment.**

(a) Proliferation of remaining human CD3^+^ T cells in NOG-IL-2/IL-15 Tg double mice. (b) Schema of *in vivo* T cell depletion by anti-CD52 antibody (MabCampath®, Alemtuzumab, Sanofi). (c, d) Frequencies of human T cells in PB (c) or spleen (d) in NOG-IL-2/IL-15 double Tg mice were determined by FACS (n=4).

**
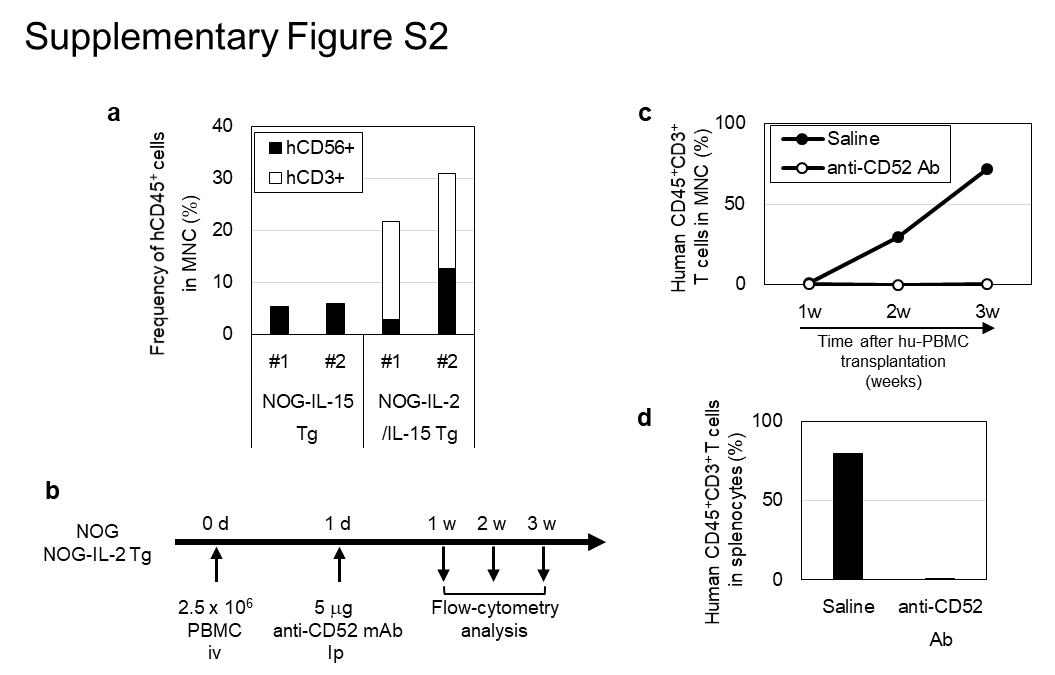
**

**Figure S3. Production of granzyme A and perforin molecules in NOG-IL-15 or NOG-IL-2/IL-15 double Tg mice.**

The staining of cells were conducted as described in Figure 5 (b)


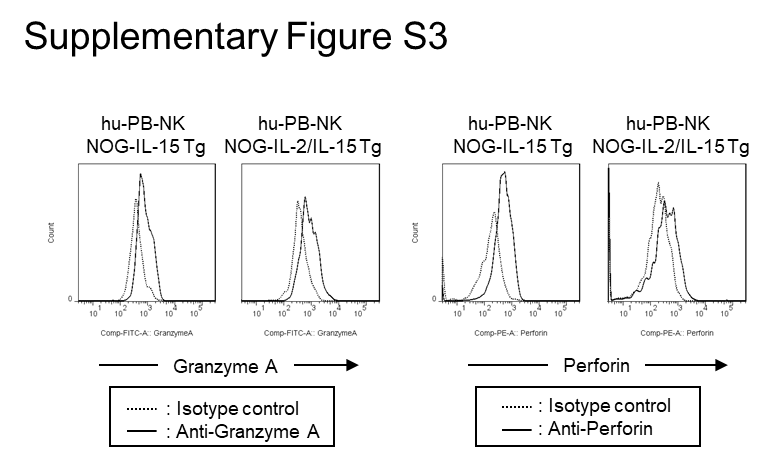


**Figure S4. *in vivo* ADCC in hu-PB-NK NOG-IL-15 Tg mice.**

(a) Schema of *in vivo* ADCC experiment

NOG-IL-15 Tg mice was transplanted with human PB-NK (2x10^6^) and L428 (1x10^7^) was subcutaneously injected in 24 hrs. Administration of anti-CCR4 antibody (50 μg) or control antibody was started 1 week after tumor inoculation and repeated twice every week for up to 5 weeks.

(b) Growth of L428 in hu-PB-NK NOG-IL-15 Tg mice

Tumor size in NOG-IL-15 Tg (n=3) or hu-PB-NK NOG-IL-15 Tg mice (n=3), which were treated with anti-CCR4 antibody or control antibody, was monitored for 6 weeks. There were no statistically significant differences in tumor size in the four experimental groups.

(c) Engraftment of human PB-NK cells in tumor-bearing hu-PB-NK NOG-IL-15 Tg mice

The number of human PB-NK cells in hu-PB-NK NOG-IL-15 Tg mice was measured by FACS using Flowcount.


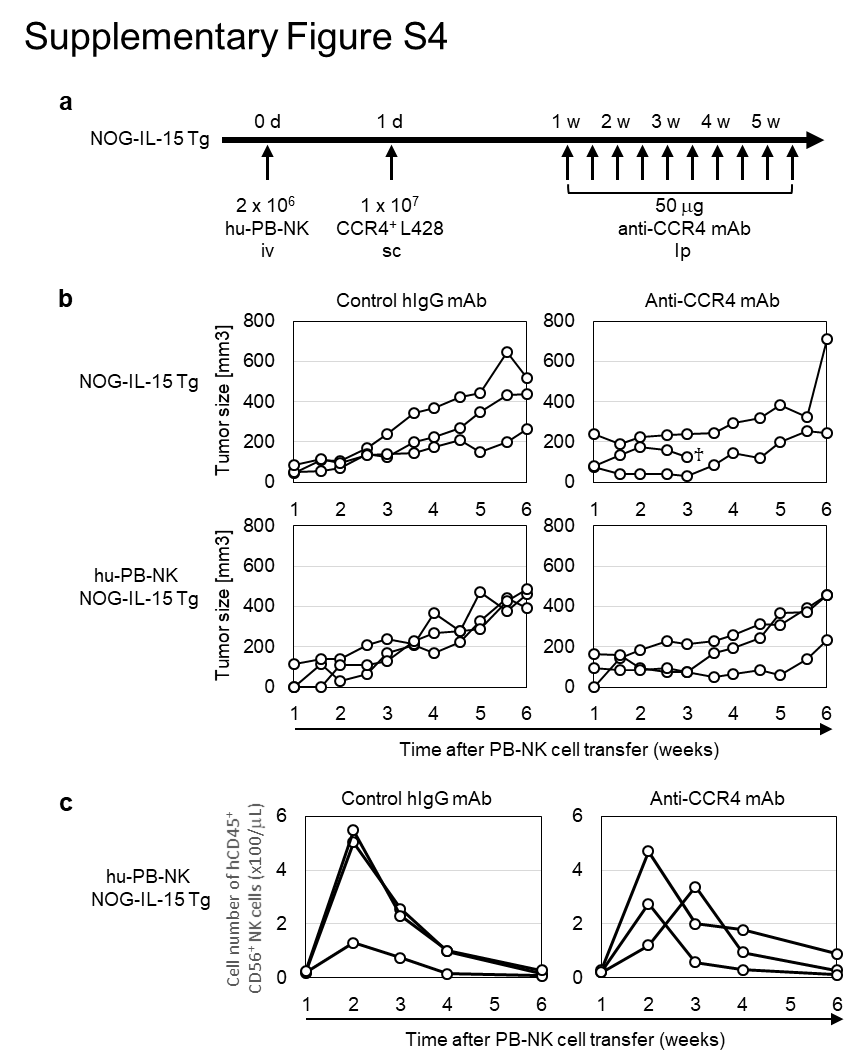


**Figure S5. Schema of *in vivo* ADCC experiment using *in vitro* expanded human NK cells**

NCI-N87 (3x10^6^) was inoculated to NOG-IL-15 Tg mice after irradiation. *In vitro* expanded NK cells (99% pure and 1x10^7^) and anti-Her2 antibody (Herceptin®, Chugai, Tokyo, Japan, 10 mg/kg) were administered by intravenous and intraperitoneal injections, respectively at the indicated time points.


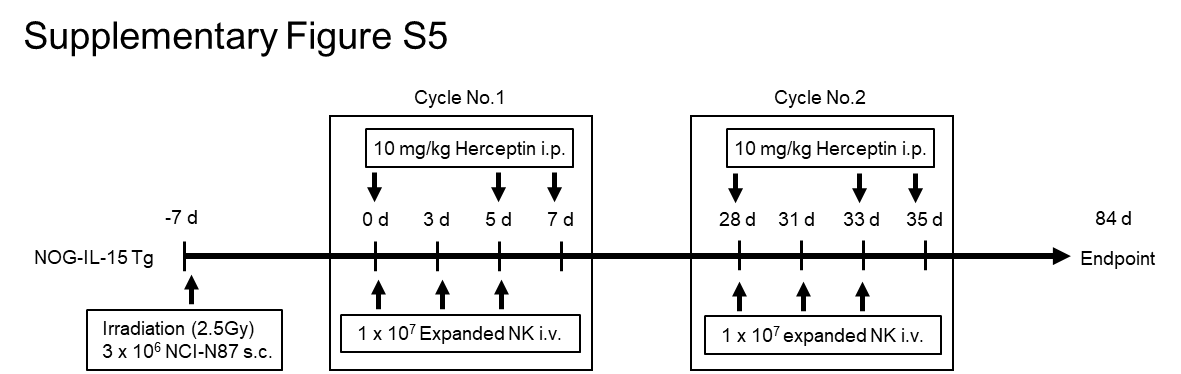


**Figure S6. Inefficient engraftment of hu-PB-NK in NOG-IL-2 Tg mice.**

**The purified NK cells (1 × 10^6^) were transferred into NOG-IL-2 Tg mice. Blood was collected and analyzed by FACS every week for 12 weeks after transfer of hu-PB-NK. The frequencies (a) and absolute numbers (b) of hu-PB-NK cells in mouse PB are shown. The results from each mouse were plotted (n = 8 for (a), n=4 (b)).**

**
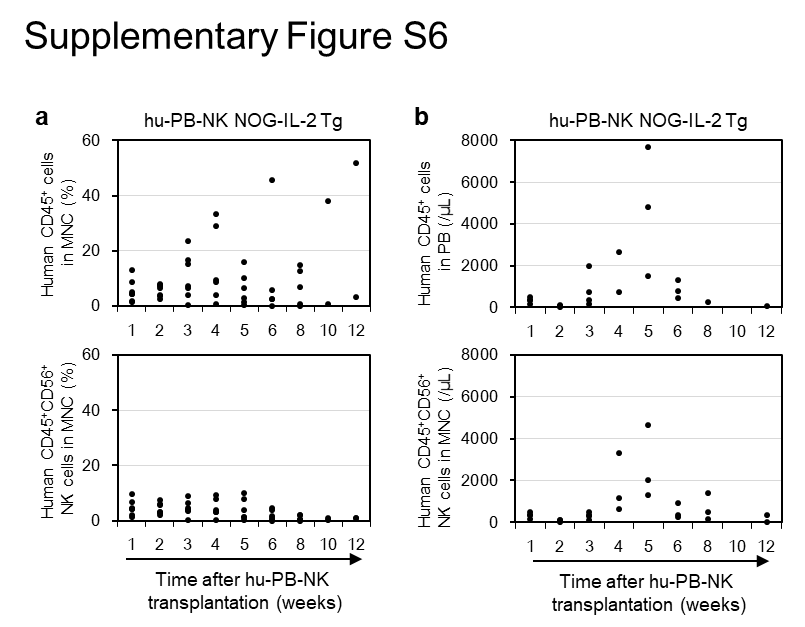
**
